# Association between leisure activities and quality of life in older adults with dementia who use day care rehabilitation services

**DOI:** 10.1002/alz.088178

**Published:** 2025-01-09

**Authors:** Masashi Kasuya, Naofumi Tanaka, Yin‐Jung Wang, Yoshitaka Ouchi

**Affiliations:** ^1^ Miyagi University, Daiwa, Miyagi Japan; ^2^ Saitama Medical University International Medical Center, Hidaka, Saitama Japan; ^3^ Tohoku Fukushi University, Sendai, Miyagi Japan; ^4^ Medical corporation Jinsenkai, Sendai, Miyagi Japan

## Abstract

**Background:**

Day care services provide not only physical exercise to improve physical function, but also a variety of activities. These activities are expected to enhance leisure activities of users and improve their QOL. However, the relationship between QOL and the presence of leisure activities in day care service users with dementia has not been clarified.

**Method:**

Participants were 76 older adults (47 women, mean age 82 ± 7 years) aged ≥65 receiving day care services with MMSE scores between 10 and 23. They were administered a series of questionnaires on leisure activities and their quality of life was assessed using the Quality of Life in Alzheimer’s Disease (QOL‐AD). Other assessments included the Geriatric Depression Scale‐15 (GDS‐15) and the Apathy Evaluation Scale (AES). The participants were divided into two groups based on whether or not they participated in leisure activities. Age, gender, nursing care level, MMSE score, GDS‐15 score, AES score, and QOL‐AD score were compared between the two groups. A covariance analysis was then conducted with leisure activity participation as the independent variable and QOL‐AD score as the dependent variable.

**Result:**

Fifty‐seven participants (37 women, age 80 ± 8 years) participated in leisure activities and 19 participants (10 women, age 83 ± 7 years) did not participate in leisure activities. There were no significant differences in age and gender between the two groups. The level of care required was significantly lower in the group with leisure activities than in the group with no leisure activities (p < 0.01), the MMSE score was significantly larger (p < 0.01) and the GDS‐15 score and AES score were significantly smaller (p < 0.05 and p < 0.01 respectively). Analysis of covariance using age, gender and nursing care level as covariates showed that the QOL‐AD score of the group with leisure activities was significantly greater than that of the group with no leisure activities (p<0.05).

**Conclusion:**

This study suggests that providing opportunities for leisure activities in day care services to older adults with dementia who do not participate in leisure activities in their daily lives may be beneficial in improving their quality of life.

